# COVID-19 in Brazil: A Message to the World

**DOI:** 10.1055/s-0041-1728660

**Published:** 2021-04-15

**Authors:** Bruno Ramalho de Carvalho

**Affiliations:** 1 Hospital Sírio-Libanês, Brasília, DF, Brazil

Dear Editor,

Although the spread and severity of the Spanish Flu pandemic in 1918 have been much more significant than the numbers of COVID-19 so far, 1 no one is able to affirm that the current crisis is nearing its end, and it is plausible to expect that it will overcome the “mother of all pandemics” and become the greatest pandemic event in human history. Such a fear is valid for the whole planet, but even more for Brazil. An observer attentive to the Brazilian scenario today, March 15, 2021, can assume exactly the reverse of the reality we would like to see: the country is considered a new global epicenter of the disease, having recorded rising curves of cases and deaths, and recently reached more than 2 thousand daily deaths. 2

The emergency of COVID-19 in Brazil is noticed all over the world and makes the country's mistakes in facing the threaten more than evident. However, the announcement of scientific inconsistency in the discourse and actions of the Brazilian government in the face of the pandemic does not come from today: native and foreign researchers have been trying, over the last year, to warn about it. 3 4 5 6 7 8 9 Furthermore, the apparent ineptitude of those who hold power is compounded by the neglect of a significant portion of the population, which, according to the mainstream media, preferred to form agglomerations from the Christmas festivities to the carnival, in spite of following the contrary recommendations from the World Health Organization. 10

Yes, what we see here is the clear effect of the so-called post-truth 11 echoing in science, mixing reason and emotion in a contradictory broth, and taking people to the extreme of a faithful belief in any speech that relieves fears or promises a miracle of healing. In September 2020, we ourselves warned about the alternation between the useful, the uncertain and the futile in the COVID-19 epidemic in Brazil, 12 where the consumption of pseudoscientific or pre-scientific information has led authorities and deniers to indisputable misunderstandings. A clear example is the defense of early treatment with hydroxychloroquine or ivermectin, 13 even after several satisfactorily well-designed studies have pointed out the ineffectiveness of those drugs, whether for prevention, early treatment or decreased mortality of people affected by SARS-CoV-2. 14 15 16 17 18 19 20 21

Not without reason, the whole world is watching closely the curves of COVID-19 in Brazil. Currently, the growth of the Brazilian epidemic is too worrying. If we are still below the United States in absolute numbers (we just passed India), 22 the relative numbers demonstrate that our epidemiological catastrophe has requirements to take the lead soon. It is worth saying here that many Brazilian citizens have been inspired by the intransigence coming from federal managers and have ignored even the basic measures to combat the transmission of the virus, such as wearing a mask and social distance. There is no way to ignore this popular behavior as a potential catalyst for this movement toward the complete lack of control of the disease.

At the moment we are dealing with the threat of new variants, 23 24 25 26 which appear to be easier to transmit and, therefore, can lead to the worsening of the situation, regardless of leading to more severe clinical conditions. Also, with the imminent collapse of the health system, which, according to the COVID-19 Observatory of the Oswaldo Cruz Foundation, had an occupancy of intensive care beds greater than 80% in 19 of the 27 federative units, in the last week. 27

Finally, we are facing a vaccination program well below the desirable for a country of continental dimensions like Brazil. Between conflicts with the National Health Surveillance Agency and resistance (at least in the beginning) to the acquisition of vaccines, the federal government does not inspire confidence in predicting the availability of immunizers quickly and in a number satisfactory to our people.

We are in the eye of the storm, with just over 4% of the population vaccinated, 28 almost two months after the start of the immunization campaign. With optimism, we will achieve the 50% in 2021. And we wait for the actions of the new minister of health, the fourth to assume the Ministry since the beginning of the pandemic, which already comes up facing the enormous challenge of controlling the Brazilian ship adrift.

This message to the world is both a vent and an appeal for the rescue of science in the conduct of COVID-19 in Brazil. Here, we have good researchers and solid scientific institutions, capable of providing proper support to such a movement. Science (and only it) will provide the answers we need and there is no doubt that denying it has been the most serious mistake of our government so far. It is time to change course. Otherwise, we will be charged by the world for something that we will not be able to reimburse.
